# Testing the application of polygenic risk scores in the transplant setting – Relevance for precision medicine

**DOI:** 10.1002/ctm2.1009

**Published:** 2022-08-15

**Authors:** Abraham Shaked, Bao‐Li Loza, Kim Olthoff, Brendan Keating

**Affiliations:** ^1^ Department of Surgery University of Pennsylvania Philadelphia Pennsylvania USA

## 1 POLYGENIC RISK SCORES FOR RISK STRATIFICATION FOR THE DEVELOPMENT OF SYSTEMIC DISEASE

Genome‐wide association studies (GWAS) have been used to identify novel variant‐trait associations and successfully identified the risk loci for multi‐gene diseases. This led to the development of polygenic risk scores (PRS) that can be used to stratify populations at risk for the development of common diseases. Recent studies using data from the UK Biobank and the USA Million Veteran's studies provided reliable PRS that can be applied to identify individuals at risk for the development of commonplace diseases such as diabetes, hyperlipidaemia and cardiovascular disorders. Integrated risk scores combine PRS with demographics, anthropometrics and clinical measurements to estimate specific disease risk based on these established risk factors.^1^ Consequently, such scores may be used to pre‐empt the natural course of the disease by providing an opportunity modifying lifestyles, implementing surveillance intervals for early disease detection, and early use of treatment modalities that better manage disease progression. Prospective validation of the utility of PRS in the clinical setting is not simple as it requires randomized studies that stretch over an extremely prolonged period of follow‐up to determine treatment effect. For example, patients who are identified to be at risk for coronary heart disease, hypertension or diabetes using PRS at age of 25 would need a follow‐up of 30 years to determine whether preemptive therapy impacts cumulative risk.^1^, ^2^

## 2 THE ADVANTAGE OF STUDYING PRS IN THE TRANSPLANT SETTING

The rapid development of metabolic syndrome complications in the transplant setting provides a more expedition and unique opportunity to test whether PRS can identify individuals at risk, set up preemptive protocols for the prevention of complications and determine whether changing the genetic environment via the introduction of the donor PRS can alter the clinical outcomes. Clinical studies using large databases demonstrated that within 12 months after transplantation, the incidence of new onset diabetes in all recipients is 15%–30%.^3^ Similar observations were reported for high prevalence soon after transplantation for hypertension, hyperlipidaemia and coronary heart disease.^4^, ^5^, ^6^ The ‘environmental variables’ that are responsible for a rapid development of these clinical complications are mainly the stress of surgery and the use of immunosuppressive drugs, many of which are known to be associated with the development of these chronic diseases (PMID: 32476781, PMID: 15093807). As not all the recipients develop the clinical expression of the disease, it was appropriate to question whether these complications are accelerated in those with high genetic risk. Knowing that immunosuppression drugs contribute significantly to these side effects, modifying immunosuppression protocols to use drug combinations and dosages that are tailored to each recipient may reduce the incidence of developing the complications. Moreover, it will allow more intense monitoring of recipient at the higher risk for early intervention.

## 3 TYPE‐2 DIABETES PRS PREDICT THE RAPID ONSET OF EARLY POST‐TRANSPLANT DIABETES

In our recent manuscript published in Nature Medicine,^7^ we aimed to determine whether well‐established type‐2 diabetes–associated PRS (T2D‐PRS)^8^ can stratify the risk exposure for developing T2D within 6–12 months after liver or kidney transplantation and whether the transplanted organs transfer donor genetic risk that would modify recipients’ risk for developing post‐transplant diabetes mellitus (PTDM). The study included liver (*n* = 1146) and kidney (*n* = 533) transplant recipients for whom both recipients’ and their paired donors’ DNA were available, and for whom we had appropriate pre‐ and post‐transplant medical records which allowed the confirmation of the new onset of T2D.

**FIGURE 1:**
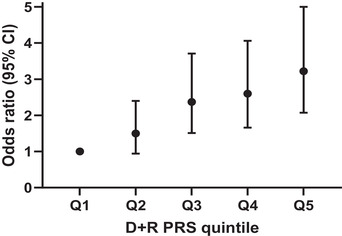
Post‐transplant diabetes mellitus (PTDM) risk among liver transplant recipients stratified by donor and recipient combined type‐2 diabetes associated polygenic risk scores (T2D‐PRS)

At first, we confirmed the association of T2D‐PRS for the diagnosis of T2D prior to transplant among kidney or liver transplant candidates. Next we tested whether T2D‐PRS are able to predict the development of PTDM in all transplant recipients (Table 1). To follow, we demonstrated that it is possible to stratify liver transplant recipients by the combined recipients and donors’ T2D‐PRS for developing PTDM (Figure 1). We proposed that dividing the entire recipient population into quintiles would allow for the design of preemptive treatment modalities that could be aimed at those at the highest risk.

**TABLE 1:** Multivariate analyses of T2D‐PRS (type‐2 diabetes associated polygenic risk scores) association with post‐transplant diabetes mellitus (PTDM), all recipients (after removing pre‐transplant diabetes)

Transplant organ	Liver		Kidney	
	(321 cases/825 controls)		(32 cases/501 controls)	
Variable	OR (95%CI)	*p*‐Value	OR (95%CI)	*p*‐Value
Recipient T2D‐PRS	1.48 (1.28–1.71)	1.3E − 07	1.9 (1.21–2.99)	.0057
Donor T2D‐PRS	1.17 (1.02–1.35)	.03	.78 (.51–1.18)	.24

*Note*: Clinical variables were considered at the time of transplantation.

## 4 THE IMPACT OF DONOR T2D‐PRS ON THE DEVELOPMENT OF POST‐TRANSPLANT DIABETES

It was equally important to determine whether donor‐specific T2D‐PRS are transferred with the donor organs. Our analyses demonstrated that kidney donors’ genetic risks do not impact the recipients’ risk for the development of T2D. However, the transplanted liver grafts do appear to carry the donors’ genetic risks into the recipients, modifying the probability of developing PTDM. This should be expected finding, considering the involvement of the liver in regulating hepatic glucose output and its crucial role in the maintenance of normal glucose homoeostasis.^9^

## 5 TESTING PREEMPTIVE TREATMENT STRATEGIES AFTER TRANSPLANTATION

The novel findings from our study set the stage for designing prospective randomized studies in the transplant setting to determine whether the application of T2D‐PRS, which was explored in the population‐level, can indeed predict outcomes in individual recipients. Studies in this area would be an excellent proof of principle of whether genetic testing can or should be used on more routine basis in any clinical setting. The high incidence and rapid expression of complications following transplantation that are predictable using PRS allows for the practical design of clinical trials to test the hypothesis in relatively small cohorts over the short time of post‐transplant follow‐up.

Cost–benefit analyses of preemptive treatment strategies should be aimed at obtaining GWAS using Single nucleotide polymorphism (SNP) arrays in all transplant candidates. This would be an achievable goal with a relatively inexpensive test that can provide sufficient genetic data to determine multiple PRS for common metabolic and cardiovascular diseases. This valuable information will allow for a personalized approach to the management of immunosuppression, such as use of a steroid‐free protocol in order to reduce the incidence of T2D, more intense monitoring of glucose levels in the higher T2D‐PRS recipients or the avoidance of mTOR inhibitors in those who are found to have higher lipid‐PRS.

Our observations could also have significant implication in the living donor setting where prospective donor genetics can be analysed before donation and may be considered part of a selection process to determine which donor has the most favourable compatibility impacting the well‐being of the recipient. For example, it may be questioned whether it is appropriate to test T2D‐ and/or lipid‐PRS in the case where two adults wish to donate a liver segment for a child, as selecting the individual with a more favourable PRS may save the child from long‐term cardiovascular complications that are transferred with the liver segment.

Our recent explorations present the potential for a unique and realistic application of PRS in prospective randomized studies in the transplant setting, and the relative short‐term follow‐up that is needed will provide an important proof of principle data to determine the utility of PRS in precision medicine.
